# Burden of care and a sense of loneliness in caregivers of children with type 1 diabetes. a cross-sectional study

**DOI:** 10.1186/s13030-023-00291-4

**Published:** 2023-10-06

**Authors:** Ewa Kobos, Sylwia Rojkowska, Alicja Szewczyk, Beata Dziedzic

**Affiliations:** 1 Department of Development of Nursing, Social and Medical Sciences, Faculty of Health Sciences, Medical University of Warsaw, Warsaw, Poland; 2 Polish Federation for Diabetes Education, Warsaw, Poland; 3 Central Clinical Hospital of the MSWiA, Warsaw, Poland; 4 Children’s Memorial Health Institute, Warsaw, Poland

**Keywords:** Type 1 diabetes, Child, Caregiver, Burden of care, Loneliness

## Abstract

**Background:**

Treatment of type 1 diabetes is a process involving not only sick children, but also their caregivers.

**Aim:**

To assess the burden of care and sense of loneliness in caregivers of children with type 1 diabetes. Also, an analysis was conducted of the connection between sociodemographic factors characterizing caregivers and the clinical factors characterizing sick children and between the burden of care and the sense of loneliness.

**Materials and methods:**

The study included 125 caregivers of children with type 1 diabetes. In order to collect the research data, the Caregiver Burden Scale and the Revised UCLA Loneliness Scale were used.

**Results:**

In the research group, the total result in the caregiving burden scale was 2.14, which remains within the average burden level. Caregivers showed the highest burden level in the General Strain Subscale. The analysis showed that mothers experience a greater burden of care than fathers in the General Strain Subscale and that caregivers of younger children are more burdened with care within the Isolation and Disappointment Subscales. Moderate high degree of loneliness was shown in 4.8% of caregivers. A higher burden of care for caregivers of children with type 1 diabetes is accompanied by a higher sense of loneliness.

**Conclusions:**

The results of this study may help healthcare professionals plan a holistic, family-centered care program that will take into account factors that increase the burden of care: younger age of the affected child, motherhood, caregiver unemployment, feelings of loneliness, lower education, caregiver unemployment, blood glucose meter measurements, and frequent night-time blood glucose measurements.

**Supplementary Information:**

The online version contains supplementary material available at 10.1186/s13030-023-00291-4.

## Background

Type 1 diabetes constitutes about 10–20% of cases of diabetes in Poland (15,220 cases < 20 years), and it is one of the most common childhood chronic diseases [1]. Worldwide, type 1 diabetes is estimated to affect 1.52 million people. In 2022, there were 530,000 new cases of T1D diagnosed at all ages, with 201,000 of these less than 20 years of age [2]. The epidemiological data indicate an increase in diabetes incidence, especially in the youngest age group. In Poland, the highest diabetes incidence occurs in children aged 2, 4, and 6 years, as well as 10–14 years [3].

The burden of care refers to how caregivers perceive their responsibilities, and the limitations they encounter also define the physical, psychological and social reactions of the caregiver, which are associated with the lack of an established balance between the care needs and other responsibilities of the caregiver. The objective burden of care is believed to be the time and effort needed to meet a patient’s needs, including financial costs, family relationships, and social functions. The subjective burden of care refers to the amount of stress caregivers are subjected to when they face the objective burden of care [4].

In paediatrics, type 1 diabetes is called a “family disease” because of the important role the family plays in the therapeutic process [5]. Challenges include adapting to a new situation, physical and emotional overload, mastering the technical aspects of care, cooperation with healthcare professionals, struggling to maintain normality in life, and seeking social support systems or maintaining interpersonal relationships [6]. Parental involvement in the child’s treatment process leads to disruptions in their life, as well as in daily family functioning [7]. Parents may feel shame, regret, guilt, anxiety, fear, and depression and report poor quality of life [8, 9].

A study evaluating the burden of care in caregivers of children and adolescents with chronic diseases showed that 8.5% of them experienced severe and 35.1% moderate burden [10]. Moderate burden of care in caregivers of chronically ill children (including those with diabetes) and the highest burden of care in the General Strain aspect were found in a study by Piran et al. The perceived burden of care was related to the age of children and their caregivers, duration of the illness and care, degree of disability of the child, number of family members, and income level [11]. A study by Kobos et al. demonstrated that the overall average burden of care for children with diabetes was within the moderate range. In general, the perceived burden of care was related to the age of the child and the occupational status of the parents [12]. In a study seeking to assess the perceived burden experienced by mothers of children with type 1 diabetes, the majority of the mothers reported a moderate to severe burden. Significant negative correlations were found between burden and physical health, social relationships, mental health, and the surrounding environment [13].

The first study in Poland on the burden of care was carried out in the year 2010, when there were no insulin pumps, pump accessories, or glucose monitoring systems for children that would be reimbursed by the National Health Fund. Parents had to bear the costs of using such equipment and, as a result, only a small number of children used it. Mothers resigning from work to care for their child were not entitled to social insurance, and the system of financial and social support from the state in caring for a sick child was limited. Technological advances in diabetes care have changed the way many patients and their caregivers cope with diabetes. Currently, the use of CGM is considered ‘standard care’ for patients with type 1 diabetes [14]. Research points to the psychological benefits associated with the use of new technologies in diabetes treatment and care (particularly CGM), and in the context of families, these include improved quality of life and family functioning as well as less stress for the caregivers. The use of automated insulin delivery systems was found to be associated with improved quality of life, reduced diabetes-related stress, and improved sleep quality in the caregivers [15].

It is not clear whether reimbursement and thus more widespread use of insulin pumps and continuous glucose monitoring in addition to caregivers remaining unemployed and currently having greater access to social benefits is significantly linked to caregiving burden and perceived loneliness. Perceived loneliness among primary caregivers of children with diabetes has only been assessed in one study, in a small group of caregivers [16].

A sense of loneliness is described as a distinctive experience of an unpleasant nature, the cause of which lies in the quantitative or qualitative impoverishment of one’s social relations. It is also understood as a discrepancy between the desired and the actual social relationships [17]. One can distinguish emotional loneliness, which is associated with a sense of isolation and social loneliness, otherwise called physical - equated with the lack of sense of belonging [18, 19]. Apart from the physical presence of other people, human beings need relationships that will provide them with a sense of security, trust, and belonging. Thus, loneliness is not only limited to people living alone, but it is also visible in people surrounded by many friends [20].

Loneliness consists of three interrelated dimensions: intimate loneliness, social loneliness, and public loneliness. Intimate loneliness refers to the absence of an important person who will offer emotional support. Social loneliness relates to family ties and friendship. In this respect, it is not the quantity of interaction that counts, but the quality of the relationship. Public loneliness means perceived isolation from a group where a person can connect with other people of a similar age, such as school [20, 21]. Loneliness can lead to mental disorders: depressive states, alcoholism, suicidal thoughts, aggressive behaviour, anxiety, and physical disorders [20].

The guidelines state that a multidisciplinary team should assess general family functioning and diabetes-related functioning, particularly at times of transition and when there may be cultural and familial difficulties in adjusting to diabetes [15].

## Methods

### Design

A cross-sectional study.

### Participants

This cross-sectional study involved 125 direct caregivers of children with type 1 diabetes. The study was conducted in two diabetology clinics at tertiary-care centres during parent-child follow-up visits between January and March 2020. We had to finish our study early because of lockdown. Caregivers were selected using the convenience sampling method. The inclusion criterion for the study included a statement that: the person concerned is the direct caregiver of the child, lives with the child, spends the most time caring for the child out of all family members, the caregiver does not have more chronically ill children, and the duration of diabetes in the child is at least one year. A total of 156 caregivers were asked to participate in the study, of which 26 refused. A total of 130 questionnaires were distributed, 5 of which were excluded from the analysis because of incorrect completion.

### Outcome measures

*Caregiver Burden Scale (CB Scale).* The Polish version of the Caregiver Burden Scale (CB Scale) was used to assess the caregiver’s burden [12]. The CB scale includes 22 questions grouped into 5 subscales: General Strain, Isolation, Disappointment, Emotional Involvement, and Environment. For each question, the caregiver answered on a scale of 1 to 4. As suggested by the author of the scale, the following categories of care burden are specified: high level (3.00–4.00), medium level (2.00-2.99) and low level (1.00-1.99) [22]. The greater the number of points a respondent receives, the higher the burden level. Previous studies have confirmed the reliability of this scale using an internal consistency assessment method [11, 22].

*Revised UCLA Loneliness Scale (R-UCLA).* The Polish version of the revised UCLA Loneliness Scale (R-UCLA) was used to assess the sense of loneliness [23]. The original version of the scale, called UCLA LS, was designed by Russell et al. [17]. The scale consists of 20 statements, respondents indicate to what extent each of the statements describes them on a 4-point scale (1 = I never feel like that, 4 = I often feel like that). The maximum score is 80 points. The total score is divided into three subscales: Belongings and Affiliation, Intimate Others and Social Others. According to Perry’s classification, the following levels of loneliness were defined: 65–80 points - a high degree; 50–64 points - a moderately high degree; 35–49 points - a moderate degree; 20–34 points - a low degree of loneliness [24].

### Statistical analysis

The Spearman’s rho correlation index (rho) was used to calculate the correlation between the variables. The Kruscal Wallis (H) test was used to check the differences between more than two independent groups. For the significant differences shown in the H-test, the Mann Whitney rank test was used to verify which groups were statistically significant. The statistical inference was carried out at a standardized degree of relevance p < 0.05.

The study was conducted in accordance with the ethical principles and the Helsinki Declaration. All eligible participants were informed about the objectives of the study. It was voluntary for the caregivers to complete the questionnaire, and they had the right to withdraw their participation in the study at any time.

## Results

### Participants’ characteristics

Table 1 illustrates the characteristics of the caregivers of children with type 1 diabetes. Women accounted for 81.6% of the study group. Over a half of the respondents (58.4%) were between 36 and 45 years of age. The average caregiver age was 39 years (SD = 6.43). The largest number of respondents (31.5%) lived in rural areas, 72.8% of the respondents were married, 50.4% of the caregivers had a tertiary education, 79.2% of the respondents were professionally active.

**Table 1:** Characteristics of caregivers participating in the study

Variables		N	%
Sex	Female	102	81.6
	Male	23	18.4
Age	23–35 years old	34	27.2
	36–45 years old	73	58.4
	46–55 years old	16	14.4
Place of residence	Village	39	31.5
	City of up to 50,000 inhabitants	34	27.4
	City of 50–100,000 inhabitants	18	14.5
	City of more than 100,000 inhabitants	33	26.6
Marital status	Single	24	19.2
	In a partnership	10	8.0
	Married	91	72.8
Education	Primary/secondary/basic vocational	13	10.4
	General secondary or vocational	49	39.2
	Higher	63	50.4
Employment status	Working	99	79.2
	Not working	26	20.8
Family Generational Status	Two-generational family	111	88.8
	Multigenerational family	14	11.2

The characteristics of the children are shown in Table 2. The largest group of children (82.4%) attended state educational institutions, whereas 9.6% of them attended integrated education. Most children received insulin via insulin pumps (81.6%). The most common method of glycaemia monitoring was the use of a glucose meter (41.6%), and the target criterion for HbA1c was met by 46.4% of the children. With 47.2% of children aged 10–14 years, the average child age was 11 years (SD = 3.55). In 56.8% of the children, the duration of the disease was 1 to 3 years, and the average duration of the disease was 4 years (SD = 3.2). The mean number of glycemic measurements in children was 14 (SD = 28.73) during the day, and 8.3 (SD = 27.72) at night. A third of the respondents (32%) declared no hypoglycaemic episodes in the child in the last 6 months. On average, respondents recorded 6.9 hypoglycaemic episodes in the last 6 months (SD = 11.36).

**Table 2:** Characteristics of children with type 1 diabetes

Variables		N	%
Age	3–5 years old	7	5.6
	6–9 years old	36	28.8
	10–14 years old	59	47.2
	15–18 years old	23	18.4
Type of educational institution the child attends	State-funded kindergarten/school, general education group/class	103	82.4
	State-funded kindergarten/school, integrated group/class	12	9.6
	Private kindergarten/school, general education group/class	8	6.4
	Does not attend any institution	2	1.6
Insulin administration method	Pen	23	18.4
	Insulin pump	102	81.6
Glycaemia monitoring method	Glucometers	52	41.6
	FGM	13	10.4
	CGM	21	16.8
	Combination of different methods	39	31.2
HbA1c	HbA1c ≤ 7%	58	46.4
	HbA1c > 7%	67	53.6
Disease duration	1–3 years	71	56.8
	4–6 years	26	20.8
	7–9 years	20	16.0
	10–14 years	8	6.4
Frequency of blood glucose measurements during the day	2–4 times	18	14.5
	5–7 times	45	36.3
	8–10 times	36	29.0
	11–16 times	14	11.3
	≥ 17 times	11	8.9
Frequency of blood glucose measurements at night	Once or not at all	25	20.2
	Twice	40	32.3
	Three times	31	25.0
	4–6 times	17	13.7
	≥ 7 times	11	8.8
Number of hypoglycaemic events in the last 6 months	No decline	40	32.0
	1–3	35	28.0
	4–7	19	15.2
	8–15	14	11.2
	≥ 16 times	17	13.6

*HbA1c - Glycated Haemoglobin, CGM - Continuous Glucose Monitoring, FGM - Flash Glucose Monitoring*

### Caregiver burden and loneliness

As shown in Table 3, the total care burden CB-scale score was 2.14 points (SD = 0.56) in the study group and ranged in the middle of the burden scale. A high burden level was found in 28.5% of the caregivers on the Disappointment Sub-Scale and in 24.8% of the caregivers on the General Strain Sub-Scale.

**Table 3:** Caregiver burden assessed on the CB-Scale

CB-Scale	M	SD	Level of burden (%)		
			Low	Medium	High
General strain	2.4	0.67	27.2	48.0	24.8
Isolation	1.81	0.77	57.6	31.2	11.2
Disappointment	2.36	0.75	31.2	40.0	28.8
Emotional involvement	1.62	0.68	68.8	23.2	8.0
Environment	1.97	0.59	48.0	43.2	8.8
Total score	2.14	0.58	41.6	50.4	8.0

*SD - standard deviation, M – mean, CB Scale - Caregiver Burden Scale*

The data in Additional file 1 shows that the mothers reported greater care burden on the General Strain and Emotional Involvement Sub-Scales as compared to the fathers. The differences were near statistical significance (p = 0.056 and p = 0.051, respectively). It was shown that non-working caregivers felt more isolated and disappointed. The method of insulin administration and glycemic monitoring did not significantly differentiate between the respondents (p > 0.05) in terms of their level of care burden. A negative correlation was shown between the age of the child and the burden within the Disappointment Sub-Scale (p = 0.048).

**Insert** Additional file 1.

Table 4 shows the results obtained by the caregivers on the R-UCLA Sense of Loneliness Scale. On average, the respondents scored 33.56 points (SD = 8.43), which indicates a low sense of loneliness. Moderate loneliness levels were shown in 34.4% of the caregivers and moderately high in 4.8% of the caregivers.

**Table 4:** Loneliness assessed on the R-UCLA scale

R-UCLA	M	SD	Level of loneliness (%)			
			Low degree	Moderate degree	Moderately high degree	High degree
Belongings and Affiliation	8.87	2.48	60.8	34.4	4.8	
Intimate Others	17.83	5.39				
Social Others	6.86	2.12				
Total score	33.56	8.43				

*SD - standard deviation, M - mean difference, R-UCLA - Revised UCLA Loneliness Scale*

Additional file 2, regarding factors related to the sense of loneliness, shows that on the Belongings and Affiliation Sub-Scale caregivers with vocational or lower education achieved significantly higher scores than those with secondary and higher education (p < 0.01); non-working caregivers scored significantly higher than the working ones. Overall, as regards the Sense of Loneliness Scale, higher values were obtained by non-working caregivers (p = 0.045). The analysis showed that the mean score on this sub-scale was higher in caregivers using glucose meters than in those using flash glucose monitoring (FGM). In this subscale, caregivers of children who had glucometer glucose monitoring achieved higher scores than did the caregivers who combined different glycaemic monitoring methods (p < 0.01) using continuous glucose monitoring (CGM) (p < 0.01). The correlation analysis showed a negative relationship between the frequency of glycaemic measurements in a child at night and the results on the Intimate Others Sub-scale (p = 0.036) or the overall score (p = 0.031).

**Insert** Additional file 2.

Table 5 illustrates the positive correlation between the caregiver burden and the sense of loneliness. Overall, the greater the burden experienced by the respondent, the higher their sense of loneliness.

**Table 5:** Correlation between the caregiver’s burden and the sense of loneliness

	Belongings and Affiliation	Intimate Others	Social Others	Total score
General strain	0.280^**^	0.237^**^	0.305^**^	0.306^**^
Isolation	0.300^**^	0.338^**^	0.294^**^	0.385^**^
Disappointment	0.266^**^	0.332^**^	0.324^**^	0.367^**^
Emotional involvement	0.125	0.236^**^	0.232^**^	0.228^*^
Environment	0.196^*^	0.235^**^	0.316^**^	0.289^**^
Total score	0.288^**^	0.330^**^	0.348^**^	0.378^**^

*p < 0.05, **p < 0.01 - correlations were statistically significant

## Discussion

This study aimed to assess the burden of care and the sense of loneliness in caregivers of children with type 1 diabetes. Our results showed that the overall average burden of care for children with diabetes was in the moderate range (2.14) and was close to the average score achieved by caregivers of chronically ill children (1.98) and of children with diabetes (1.95), caregivers of children with diabetes (2.4), and caregivers of children undergoing chemotherapy (2.02) [11, 12, 25]. This study demonstrated that the highest level of caregiver burden occurs on the General Strain and Disappointment Sub-Scales. The values obtained on both subscales are within the range indicating an average burden. The General Strain subscale refers to caregivers experiencing physical and emotional disorders caused by caring activities, problems with care and the time required to provide care services. The Disappointment Sub-Scale includes experiencing financial sacrifices due to childcare; the caregiver’s expectations about their current life; the conviction that life has treated them unfairly; the caregiver’s opinion that caring for a sick child is physically demanding; and a sense of loneliness and isolation due to the child’s illness.

The greatest burden of caring for children undergoing chemotherapy was shown on the Isolation Sub-Scale, whereas in a group of caregivers of chronically ill children it was on the General Strain and Environment Sub-Scales [25]. A comparison of different chronic diseases showed the maximum burden of care among caregivers of children with cerebral palsy [11].

When analyzing the results of the present study and the one conducted in the year 2010, it is important to highlight the similar values of overall care burden as well as of General strain (2.4 vs. 2.41) and Disappointment (2.36 vs. 2.31), despite the fact that in our study approximately 60% of the children used continuous glucose monitoring and that the inclusion criteria for caregivers differed [11]. It would seem that the burden of care now as compared to the 2010 study results should be much lower. In this study, 41.6% of the children were still using a glucometer to measure their blood glucose and about 20% were administering insulin with an insulin pen injector. The data confirm that the use of diabetes technology may decrease some of the burden of care for the child. The use of an insulin pump may alleviate some of the distress and limitations experienced by caregivers: no need to administer basal insulin at particular times of the day, greater flexibility associated with feeding the child due to the ease of administering the insulin dose with the pump, and less anxiety associated with leaving the child in the care of others. The use of continuous glucose monitoring makes it easier for caregivers to make treatment decisions [26]. Currently, the widespread availability of reimbursement for insulin pumps, FGM, CGM, and new types of insulin should make it easier for caregivers to care for a sick child. Data show that most T1DM patients and their caregivers benefit from the use of new technologies in diabetes management [27, 28]. On the other hand, their use has been shown to be costly and not always refundable. Moreover, alarm fatigue, technical failures, and accuracy problems limit their use [29]. Despite the high percentage of insulin pump use, the use of CGM and FGM remains low [1, 29].

Diabetes imposes a number of new responsibilities on caregivers, such as day and night glycaemic monitoring [8]. Research shows that caregivers experience sleep deprivation and also worry about their child’s glycaemic levels at night [30–32]. A great number of responsibilities and the risk of nocturnal hypoglycaemia contribute to poor sleep quality and short sleep time [33]. Night measurements are positively correlated with the anxiety level of the parent and associated with a higher burden of care while the occurrence of hypoglycaemia is associated with emotional distress, mostly in the mothers [12, 34, 35]. Our study did not support the link between the number of night glycaemic measurements and emotional involvement, although this relationship was suggested by Kobos & Imiela in an earlier study [12]. On the other hand, the number of glycaemic measurements has been negatively correlated with the general sense of loneliness and loneliness on the Intimate Others Sub-Scale.

It is common for mothers to give up work to care for the child, receive carer giver’s benefits, and have these years included in the employment period. Research shows that families with lower socio-economic status experience higher financial burden and that the average burden of caregivers of chronically ill children and young people varies significantly in terms of their education as well as professional and material status [10, 36, 37]. Zatorska-Zoła showed that as many as 37% of the parents feel a great financial burden associated with the treatment of the child, and 46.4% families reported moderate to severe financial losses [8, 38]. Parents bear significant costs associated with the purchase of insulin and equipment for its administration or glycaemic monitoring. According to a study by Cunningham et al. the greatest burden of care is experienced by mothers over the age of 41, with primary school education, having a chronic disease, and for whom the child’s diabetes was diagnosed 3–4 years previously [39]. The literature points out that having a higher education increases parental confidence in coping with a child’s health problems [40].

According to Piran et al., employed and unemployed caregivers experience the same care burden, while in another study, unemployment increased the burden of care on the Isolation Sub-Scale [11, 25]. In our study, there were substantially fewer caregivers unemployed (n = 20.8%) than in the 2010 study (n = 53%); however, this also confirmed that this factor is associated with a higher score in overall burden and on the Isolation and Disappointment Sub-Scales [12]. The present study and the one from 2010 have confirmed that it is the caregivers of younger children who are more burdened with care in the Disappointment Sub-Scale [12]. Younger children require constant attention. Their greater dependence on parents means that caregivers spend more time caring for them. This limits their social activity and prevents them from achieving their life goals. The authors of the study assessing parental perception of the burden of care for very young children with T1DM have demonstrated that this is a widespread phenomenon which can be reduced through tailored educational programs that increase parental knowledge and that create confidence in themselves and in secondary caregivers [41].

Staying at home and focusing on the needs of a sick child can foster a sense of isolation from one’s family and friends and promote loneliness. This study showed a lower score on the Isolation Sub-Scale compared to studies performed in 2010 [12]. With the duration of the disease, parents and their children try to lead normal lives or participate in social life, and caregivers delegate responsibility for diabetes self-management to their children [42, 43]. New glycaemic monitoring technologies enable parents to support their children in distance self-care [15].

Research indicates that the mothers of children with type 1 diabetes should receive psychological support to better cope with the burden of care. The International Society for Pediatric and Adolescent Diabetes strongly recommends providing psychological support not only for the sick children but also for their parents. Evidence-based interventions for caregivers/parents to reduce stress and increase resilience, increase social support, promote parental involvement in care, goal setting and problem solving therapy for family problems and conflicts, and cognitive behavioural therapy are important in clinical care [15, 44]. Research suggests that caregivers of children and adolescents with T1D need the help of a treatment team in health care, mental health, social support, and family management of childhood diabetes [45].

Mothers who spend most of their time on treatment and childcare activities see this process as demanding [46]. However, the lack of freedom experienced by the mothers may result from difficulties in sharing responsibilities with other family members [35]. This study shows that the mothers experienced a greater burden of caring for a sick child compared to the fathers and that they are more emotionally involved in the treatment process. Studies of other authors have also confirmed a higher overall burden of care for chronically ill children (general strain) on the part of the mothers, as compared to the fathers [11]. This may also involve, on the one hand, the low involvement of the fathers in the care of a child with diabetes, and on the other hand, the conviction of the mothers that they will take better care of the child than the fathers who are thus often excluded from caring for the child [47, 48]. Parents also report numerous burdens associated with other people caring for their sick child. These include difficulties in finding people who could care for the child; limited trust for secondary caregivers in childcare; the constant burden of vigilance over the care of the child, even when it was under the care of another adult guardian [41]. The withdrawal of fathers from childcare often leads to a weakening of communication between the spouses [47]. Being a direct caregiver for a child with type 1 diabetes is associated with a life of constant readiness, and it brings out emotional consequences, a feeling of constant exhaustion, and lack of rest [43].

According to research, the mothers of children with chronic diseases show greater loneliness than the mothers of healthy children [16]. The average UCLA score among the mothers of chronically ill children was 36.11, higher than that of the mothers of healthy children − 29.76. In this study, about a third of caregivers demonstrated a sense of loneliness at an average level. It was lower compared to that of the mothers of chronically ill children and higher compared to that of the mothers of healthy children [16].

A study using a multidimensional self-report questionnaire (including the Loneliness Sub-Scale) that aimed to compare the incidence of disease-related distress symptoms in the parents of children with cancer and those with diabetes showed that the parents of children with cancer reported a greater sense of loneliness [49]. The distress levels of the parents of cancer patients significantly exceeded those of the parents of diabetic patients as regards loneliness. However, as the authors of the study in paediatric diabetes point out, the persistence or intensification of distress over time is of specific clinical relevance. In this study, the feelings of loneliness were not significantly lower in the caregivers of older children or children with a longer duration of the disease. Research suggests that sharing responsibility is important for the development of self-management in adolescents with diabetes. One study found that parents generally stay responsible longer when adolescents follow a continuous subcutaneous insulin (CSII) regimen compared to multiple injections per day. A qualitative analysis has shown that new technologies increase the number and complexity of responsibility-sharing arrangements and that parents have a constant sense of responsibility for the treatment of their children, facing new challenges when their children enter the teenage and adolescent periods [42].

A higher sense of loneliness on the Belongings and Affiliation Sub-Scale was demonstrated by caregivers whose children monitored glycaemia using a glucose meter. The overall feeling of loneliness was greater with the increase in the number of glycaemic measurements at night. There is evidence suggesting that caregivers, in order to reduce their concerns about nocturnal hypoglycaemia, regularly check blood glucose levels throughout the night, which leads to exhaustion and chronic sleep disturbances [26]. Caregivers feel tired from night glycaemic measurements, which can make them feel lonely. Research shows that caregivers focusing on the performance of new diabetes-related duties for a child neglect the relationship with their spouse, family, or other children [50] and that ties with their offspring weaken [51, 52]. These situations lead to a sense of loneliness [43]. In a group of mothers of chronically ill children, a significant inverse relationship was observed between loneliness and social support [16].

The results of this study allow for a better understanding of how type 1 diabetes affects the caregivers of a sick child. In the planning of the care of a child with diabetes, healthcare professionals should take into account family-oriented interventions to reduce the burden and related care. The care burden in immediate caregivers of children with chronic diseases is defined and structured based on the psychosocial and sociodemographic profile of the family and the caregiver [53]. This study implies that medical staff should be encouraged to assess the parents of children with T1D from a psychosocial perspective. The need to share family responsibilities should be addressed in the education of parents of children with type 1 diabetes. Diabetes education of caregivers and their re-education should include not only the mother but also other family members. Educational programmes should include information on the availability of medical technologies, sources of reimbursement or funding, and the advantages and disadvantages of their use, so that parents can make informed decisions. Outside of the family, the child’s surrounding environment should be ready to provide care for a small child with diabetes in order to relieve the caregiversˈ burden and make it easier for them to have a job and social relationships.

This study provides evidence for further research and implementation of family intervention strategies based on an assessment of these factors.

### Limitations

Our study has some limitations. A small number of caregivers took part in the study; convenience sampling was used, which means that only available parents were enrolled in the study. Thus, there is limited scope to make generalisations and conclusions about the general population of caregivers. In order to increase the likelihood of being equally likely to become part of the study group, each week several caregivers meeting the inclusion criteria were asked to participate in the study; these were caregivers of children with follow-up appointments with different diabetologists.

In Poland, mothers are much more likely to look after their children and give up or limit their working hours in order to be able to care for a sick child. Research mainly reflects the observations of mothers, thus future research should aim to take into account the observations of fathers and other caregivers. The study participants were caregivers of children with diabetes who were treated in state polyclinics. Caregivers using private institutions may have different socio-economic characteristics and thus the results of this study cannot be generalized to all caregivers.

The literature review shows that there is no data to compare the current findings of loneliness in caregivers of children with diabetes. Therefore, the results of this study, in this respect, should be considered preliminary and further studies should be undertaken. Due to the COVID-19 pandemic, the study finished early. In the future, we plan to extend the study to other centres specialising in the treatment of children with diabetes and to include more caregivers in the study.

## Conclusion

The direct caregivers of children with diabetes generally show an average level of care and a low sense of loneliness. The caregivers of younger diabetic children, as well as mothers and caregivers who remain unemployed, should be under more frequent monitoring for the burden of care. Lower education, caregiver unemployment, blood glucose measurements, and more frequent night-time blood glucose measurements are risk factors for increased loneliness among caregivers.

If the perceived loneliness of the primary caregiver is greater when they have a lower education, are unemployed, when the child is monitored with a glucose meter, or when nighttime glycaemic readings are frequent; it may be important to involve the caregiver and another family member (who can take over some of the caregiving responsibilities) in the education and re-education process to reduce this feeling. Also, familiarity with diabetes self-monitoring guidelines should be assessed regularly, the use of new technologies in blood glucose monitoring should be discussed, and an assessment should be made of the fear of nocturnal hypoglycaemic events, which the literature suggests may be the cause of nocturnal multiple blood glucose measurements in the child.

The training of caregivers with lower education to care for the child should also be tailored to their abilities and there should be more frequent evaluation of the caregiver’s management of the child’s diabetes. Lower education and being unemployed are factors that decrease a family’s financial status. It is important to educate this group of caregivers about the sources of social and financial support.

### Electronic supplementary material

Below is the link to the electronic supplementary material.

Supplementary Material 1

## Data Availability

The datasets used and/or analyzed in the current study are available from the corresponding author upon reasonable request.

## Abbreviations

T1D: Type 1 Diabetes
CB Scale: Caregiver Burden Scale
R-UCLA: Revised UCLA Loneliness Scale

## References

[1] National Health Fund. (2019). National Health Fund – pro-health series: Diabetes https://zdrowedane.nfz.gov.pl/pluginfile.php/205/mod_resource/content/4/nfz_o_zdrowiu_cukrzyca.pdf (Access: 10.14.2023).

[2] Ogle GD, Wang F, Gregory GA, Maniam J. Type 1 diabetes estimates in children and adults – 2022. IDF Atlas Reports. www.diabetesatlas.orgInternational (Access: 14.04.2023).

[3] Niechciał E, Skowrońska B, Michalak M, Fichna P (2018). Ketoacidosis at diagnosis of type 1 diabetes in children and adolescents from Wielkopolska province in Poland: prevalence, risk factors and clinical presentation. Clin Diabetol.

[4] Liu Z, Heffernan C, Tan J (2020). Caregiver burden: a concept analysis. Int J Nurs Sci.

[5] Moore SM, Hackworth NJ, Hamilton VE, Northa EP, Cameron FJ (2013). Adolescents with type 1 diabetes: parental perceptions of child health and family functioning and their relationship to adolescent metabolic control. Health Qual Life Outcomes.

[6] Dhada BL, Blackbeard DR (2019). Caregivers of children with diabetes mellitus: challenges of caring for and perceptions of consultations in a south african public sector context. South Afr Family Pract.

[7] Kobos E, Imiela J, Lenczuk-Gruba A (2017). Diabetes, child care, and performance of family functions. Med Stud.

[8] Zatorska, –, Zoła MB (2018). Challenges for parents of children with diabetes. Piel Zdr Publ.

[9] Pate T (2016). Families of children with chronic illness and the relational family model. The Person and the Challenges.

[10] Adib-Hajbaghery M, Ahmadi B (2019). Caregiver Burden and its Associated factors in caregivers of children and adolescents with chronic conditions. Int J Community Based Nurs Midwifery.

[11] Piran P, Khademi Z, Tayari N, Mansouri N (2017). Caregiving burden of children with chronic diseases. Electron Physician.

[12] Kobos E, Imiela J (2015). Factors affecting the level of burden of caregivers of children with type 1 diabetes. Appl Nurs Research: ANR.

[13] Gallegos E, Harmon KB, Lee G, Qi Y, Jewell VD (2023). A descriptive study of the quality of life and burden of mothers of children and adolescents with type 1 diabetes. Occup Ther Health Care.

[14] Holt RIG, DeVries JH, Hess-Fischl A, Hirsch IB, Kirkman MS, Klupa T, Ludwig B, Nørgaard K, Pettus J, Renard E, Skyler JS, Snoek FJ, Weinstock RS, Peters AL (2021). The management of type 1 diabetes in adults. A Consensus Report by the american Diabetes Association (ADA) and the European Association for the study of diabetes (EASD). Diabetes Care.

[15] de Wit M, Gajewska KA, Goethals ER, McDarby V, Zhao X, Hapunda G, Delamater AM, DiMeglio LA (2022). ISPAD Clinical Practice Consensus Guidelines 2022: psychological care of children, adolescents and young adults with diabetes. Pediatr Diabetes.

[16] Florian V, Krulik T (1991). Loneliness and social support of mothers of chronically ill children. Soc Sci Med.

[17] Russell D, Peplau LA, Cutrona CE (1980). The revised UCLA Loneliness Scale: concurrent and discriminant validity evidence. J Pers Soc Psychol.

[18] Yanguas J, Pinazo-Henandis S, Tarazona-Santabalbina FJ (2018). The complexity of loneliness. Acta Bio-Medica: Atenei Parmensis.

[19] Xia N, Li H, Loneliness (2018). Social isolation, and Cardiovascular Health. Antioxid Redox Signal.

[20] Cacioppo S, Grippo AJ, London S, Goossens L, Cacioppo JT (2015). Loneliness: clinical import and interventions. Perspect Psychol Science: J Association Psychol Sci.

[21] Dunbar RIM (2014). The Social Brain: psychological underpinnings and implications for the structure of Organizations. Curr Dir Psychol Sci.

[22] Elmståhl S, Malmberg B, Annerstedt L (1996). Caregiver’s burden of patients 3 years after stroke assessed by a novel caregiver burden scale. Arch Phys Med Rehabil.

[23] Kwiatkowska MM, Rogoza R, Kwiatkowska K (2017). Analysis of the psychometric properties of the revised UCLA Loneliness Scale in a polish adolescent sample. Curr Issues Personality Psychol.

[24] Perry G (1990). Loneliness and coping among tertiary level adult cancer patients in the home. Cancer Nurs.

[25] Rubira EA, Marcon SR, Belasco AGS, Gaíva MAM, Espinosa MM (2020). Burden and quality of life of caregivers of children and adolescents with chemotherapy treatment for cancer. Acta Paul Enferm.

[26] Kimbell B, Lawton J, Boughton C, Hovorka R, Rankin D (2021). Parents’ experiences of caring for a young child with type 1 diabetes: a systematic review and synthesis of qualitative evidence. BMC Pediatr.

[27] Tauschmann M, Hovorka R (2018). Technology in the management of type 1 diabetes mellitus - current status and future prospects. Nat Rev Endocrinol.

[28] Mueller-Godeffroy E, Vonthein R, Ludwig-Seibold C, Heidtmann B, Boettcher C, Kramer M, Hessler N, Hilgard D, Lilienthal E, Ziegler A, Wagner VM, German Working Group for Pediatric Pump Therapy (agip) (2018). Psychosocial benefits of insulin pump therapy in children with diabetes type 1 and their families: the pumpkin multicenter randomized controlled trial. Pediatr Diabetes.

[29] Barnard K, Crabtree V, Adolfsson P, Davies M, Kerr D, Kraus A, Gianferante D, Bevilacqua E, Serbedzija G (2016). Impact of type 1 Diabetes Technology on Family Members/Significant others of people with diabetes. J Diabetes Sci Technol.

[30] Majidi S, ODonnel H, Benson K, Driscoll KA (2018). Clinic-wide screening of fear of hypoglycemia in mothers and fathers of children with type 1 diabetes. Diabetes.

[31] Abitbol L, Palmert MR (2021). When low blood sugars cause high anxiety: fear of Hypoglycemia among parents of Youth with type 1 diabetes Mellitus. Can J Diabetes.

[32] Carreon SA, Cao VT, Anderson BJ, Thompson DI, Marrero DG, Hilliard ME (2022). I don’t sleep through the night’: qualitative study of sleep in type 1 diabetes. Diabet Med.

[33] Feeley CA, Sereika SM, Chasens ER, Siminerio L, Charron-Prochownik D, Muzumdar RH, Viswanathan P (2021). Sleep in parental caregivers and children with type 1 diabetes. J Sch Nurs.

[34] Macaulay GC, Boucher SE, Yogarajah A, Galland BC, Wheeler BJ (2020). Sleep and night-time caregiving in parents of children and adolescents with type 1 diabetes Mellitus - A qualitative study. Behav Sleep Med.

[35] Haugstvedt A, Wentzel-Larsen T, Rokne B, Graue M (2011). Perceived family burden and emotional distress: similarities and differences between mothers and fathers of children with type 1 diabetes in a population-based study. Pediatr Diabetes.

[36] Tong H, Qiu F, Fan L (2022). Characterising common challenges faced by parental caregivers of children with type 1 diabetes mellitus in mainland China: a qualitative study. BMJ Open.

[37] Lindley LC, Mark BA (2010). Children with special health care needs: impact of health care expenditures on family financial burden. J Child Fam stud.

[38] Dehn-Hindenberg A, Saßmann H, Berndt V, Biester T, Heidtmann B, Jorch N, Kim-Dorner SJ, Konrad K, Lilienthal E, Nellen-Hellmuth N, Neu A, Ziegler R, Lange K (2021). Long-term Occupational Consequences for families of children with type 1 diabetes: the mothers take the Burden. Diabetes Care.

[39] Cunningham NR, Vesco AT, Dolan LM, Hood KK (2011). From caregiver psychological distress to adolescent glycemic control: the mediating role of perceived burden around diabetes management. J Pediatr Psychol.

[40] Medway M, Tong A, Craig JC, Kim S, Mackie F, McTaggart S, Walker A, Wong G (2015). Parental perspectives on the financial impact of caring for a child with CKD. Am J Kidney Diseases: Official J Natl Kidney Foundation.

[41] Commissariat PV, Harrington KR, Whitehouse AL, Miller KM, Hilliard ME, Van Name M, DeSalvo DJ, Tamborlane WV, Anderson BJ, DiMeglio LA, Laffel LM (2020). I’m essentially his pancreas: parent perceptions of diabetes burden and opportunities to reduce burden in the care of children < 8 years old with type 1 diabetes. Pediatr Diabetes.

[42] Gardener L, Desha L, Bourke-Taylor H, Ziviani J (2022). Responsibility sharing for adolescents with type 1 diabetes: a scoping review. Chronic Illn.

[43] Iversen AS, Graue M, Haugstvedt A, Råheim M (2018). Being mothers and fathers of a child with type 1 diabetes aged 1 to 7 years: a phenomenological study of parents’ experiences. Int J Qualitative Stud Health Well-being.

[44] Eccleston C, Fisher E, Law E, Bartlett J, Palermo TM (2015). Psychological interventions for parents of children and adolescents with chronic illness. Cochrane Database Syst Rev.

[45] Zhao X, Ai Z, Chen Y, Wang J, Zou S, Zheng S (2019). The effectiveness of parenting interventions on Psychosocial Adjustment in parents of children and adolescents with type 1 diabetes: a Meta-analysis. Worldviews Evid Based Nurs.

[46] Keklik D, Bayat M, Başdaş Ö (2020). Care burden and quality of life in mothers of children with type 1 diabetes mellitus. Int J Diabetes Dev Ctries.

[47] Zamarlik MA (2019). The degree of fathers’ involvement in taking care of children with diabetes and its implications in family functioning in the assessment of diabetic children’s mothers. Pediatr Endocrinol Diabetes Metab.

[48] Makara-Studzińska M, Somasundaram S, Ashraf GM, Gogacz M, Madej A, Izydorczyk B, Leszek J, Lebedeva SA, Chubare VN, Tarasov VV, Kirkland E, Aliev G. Assessment of Psychosocial Functioning of mothers of children with diabetes Mellitus compared to mothers of healthy children. Biomed Res Int. 2019;6821575. 10.1155/2019/6821575. 10.1155/2019/6821575 PMC6481141 31093501

[49] Boman KK, Viksten J, Kogner, Samuelsson U. Serious illness in childhood: the different threats of cancer and diabetes from a parent perspective. The Journal of Pediatrics 2004; 145(3):373–379. 10.1016/j.jpeds.2004.05.043 15343194

[50] Nieuwesteeg AM, Hartman EE, Aanstoot HJ, vanBakel HJ, Emons WH, van Mil E, Pouwer F. The relationship between parenting stress and parent-child interaction with health outcomes in the youngest patients with Type 1 diabetes (0–7 years). European Journal of Pediatrics 2016; 175:329–338. 10.1007/s00431-015-2631-4 PMC4757610 26438336

[51] Chan KKL, Shorey S (2022). Experiences and needs of children with siblings diagnosed with type 1 diabetes: a mixed studies systematic review. J Pediatr Nurs.

[52] Havill N, Fleming LK, Knafl K (2019). Well siblings of children with chronic illness: a synthesis research study. Res Nurs Health.

[53] Toledano-Toledano F, Domínguez-Guedea MT (2019). Psychosocial factors related with caregiver burden among families of children with chronic conditions. Biopsychosoc Med.

