# Supplementary material for: Burden of care and a sense of loneliness in caregivers of children with type 1 diabetes. a cross-sectional study
Source: Biopsychosoc Med. 2023 Oct 6;17:34. doi: 10.1186/s13030-023-00291-4 (PMC10559508; doi:10.1186/s13030-023-00291-4)
Supplement: Supplementary file 1 — Supplementary Material 1 [file 13030_2023_291_MOESM1_ESM.docx]

**Additional file 1.** Factors related to the caregiver, the child and the burden of care

| **Factors** | | | **General Strain** | **Isolation** | **Disappointment** | **Emotional involvement** | **Environment** | **Total score** |
| --- | --- | --- | --- | --- | --- | --- | --- | --- |
| Factors associated with caregivers | Sex | U  p | 874.0  .056 | 1071.0  .508 | 1086.5  . 580 | 875.0  .051 | 1073.5  .519 | 1002.5  .224 |
|  | Age | rho  p | -0.040  .660 | -0.050  .583 | -0.106  .239 | -0.119  .186 | -0.135  .132 | -0.097  . 281 |
|  | Place of residence | H  p | 4.37  .224 | 4.05  .256 | 3.21  .361 | 2.62  .454 | 1.46  .691 | 3.77  .287 |
|  | Marital status | U  p | 1021.0  .230 | 1090.0  .436 | 1133.5  .621 | 1147.0  .675 | 1127  .543 | 1022.0  .183 |
|  | Education | H  p | 0.41  .817 | 3.47  .177 | 1.11  .575 | 0.27  .872 | 0.28  .868 | 0.33  .849 |
|  | Employment status | U  p | 993.5  .074 | 980.0  .057 | 968.0  .051 | 1203.5  .602 | 1051.0  .144 | 997.5  .049* |
| Factors associated with children | Insulin administration method | U  P | 1072.5  .521 | 1050.5  .427 | 1081.0  .556 | 981.5  .210 | 1140.0  .831 | 981.5  .172 |
|  | Blood glucose monitoring method | H  p | 0.41  .937 | 0.70  .873 | 2.30  .513 | 3.14  .370 | 0.82  .845 | 1.11  .775 |
|  | Age of the child | rho  p | -0.161  .073 | -0.165  .066 | -0.177  .048* | -0.058  .521 | -0.159  .077 | -0.195  .030* |
|  | Duration of the disease | rho  p | -0.024  .793 | 0.082  .366 | 0.015  .871 | 0.021  .812 | -0.126  .162 | -0.035  .701 |
|  | Average measurement frequency during the day | rho  p | 0.040  .662 | 0.034  .710 | 0.096  .287 | -0.104  .250 | -0.027  .766 | 0.081  .373 |
|  | Average measurement frequency at night | rho  p | 0.058  .521 | -0.061  .504 | 0.011  .903 | 0.027  .770 | -0.009  .917 | 0.081  .371 |
|  | Glycated haemoglobin level | rho  p | 0.060  .504 | 0.128  .155 | -0.024  .789 | 0.081  .370 | 0.052  .561 | 0.117  .167 |
|  | Number of hypoglycaemic events in the last 6 months | rho  p | 0.072  .427 | 0.075  .406 | 0.075  .406 | 0.119  .187 | 0.059  .512 | 0.136  .132 |

*H - Kruskal-Wallis test, U - Mann Whitney test, rho - Spearman's Correlation Coefficients, p - statistical significance, *p< .05 - correlations were statistically significant, R-UCLA - Revised UCLA Loneliness Scale*

**Additional file 2.** Factors related to the caregiver and child versus sense of loneliness

| Factors | | | R-UCLA Scale | | | |
| --- | --- | --- | --- | --- | --- | --- |
|  |  |  | Belongings and Affiliation | Intimate Others | Social Others | Total score |
| Factors associated with caregivers | Sex | U  p | 1141.0  .836 | 1128.0  .774 | 1085.0  .563 | 1160.0  .934 |
|  | Age | rho  p | 0.013  .882 | -0.034  .705 | -0.012  .893 | -0.029  .750 |
|  | Place of residence | H  p | 4.67  .198 | 0.86  .836 | 0.81  .847 | 0.67  .880 |
|  | Marital status | U  p | 1165.0  .765 | 1030.0  .253 | 979.5  .133 | 1133.0  .620 |
|  | Education | H  p | 12.04  .002* | 2.02  .364 | 1.74  .418 | 4.80  .091 |
|  | Employment status | U  p | 691.5  .000* | 1078.5  .204 | 1275.5  .943 | 957.5  .045* |
| Factors associated with children | Insulin administration method | U  p | 948.0  .146 | 1166.5  .967 | 1075.0  .520 | 1132.0  .794 |
|  | Glycaemia monitoring method | H  p | 14.52  .002* | 2.11  .550 | 0.31  .959 | 4.01  .260 |
|  | Age of the child | rho  p | -0.077  .396 | 0.010  .912 | -0.064  .479 | -0.031  .733 |
|  | Duration of the disease | rho  p | .028  .761 | -0.080  .376 | -0.046  .611 | -0.054  .551 |
|  | Average measurement frequency during the day | rho  p | -0.075  .406 | -0.106  .241 | -0.119  .190 | -0.107  .238 |
|  | Average measurement frequency at night | rho  p | -0.093  .304 | -0.188  .036* | -0.168  .062 | -0.194  .031* |
|  | Glycated haemoglobin level | rho  p | 0.121  .178 | -0.086  .338 | -0.065  .471 | -0.038  .674 |
|  | Number of hypoglycaemic events in the last 6 months | rho  p | -0.044  .630 | 0.013  .886 | -0.094  .296 | -0.026  .771 |

*H - Kruskal-Wallis test, U - Mann Whitney test; rho - Spearman's Correlation Coefficients, p - statistical significance, *p<0.05 - correlations were statistically significant, R-UCLA - Revised UCLA Loneliness Scale*
